# The Future Health Workforce: Integrated Solutions and Models of Care

**DOI:** 10.3390/ijerph18062849

**Published:** 2021-03-11

**Authors:** Madhan Balasubramanian, Stephanie Short

**Affiliations:** Faculty of Medicine and Health, The University of Sydney, Sydney 2006, Australia; stephanie.short@sydney.edu.au

The health workforce is a vital aspect of health systems, both essential in improving patient and population health outcomes and in addressing contemporary challenges such as universal health coverage (UHC) and sustainable development goals (SDGs). There is an increasing body of research that indicates that if the health workforce were to be redesigned from the ground up—based on population needs—we would see a very different configuration of the health workforce. This makes us wonder how one could design or develop innovative health workforce solution(s) for the future in order to make the health workforce more responsive to population needs.

The 21st century presents several challenges to the health workforce and the health professions that require thoughtful consideration and analysis. Health inequalities continue to exist both within and across countries, especially affecting vulnerable and disadvantaged groups. Disease patterns are changing, with a rise in chronic conditions and non-communicable diseases, the COVID-19 pandemic notwithstanding. Increased life expectancies also present us with the challenge of meeting care provisions for an ageing population. Workforce shortages, geographic maldistribution, and international migration are omnipresent.

Health workforce solutions have been diverse and generally dependent on condition, context, or country-specific scenarios. New health occupations, as well as reforming the scopes of practice of existing occupations, have been widely debated as solutions. Of importance has been how different health personnel groups can work collaboratively as a team, and at different levels of care—primary, secondary, and tertiary. Models of care specific to population groups (e.g., Indigenous peoples, children, or older people) as well as health conditions (e.g., cancer or oral health), and health strategies (e.g., rehabilitation) are emerging, with varied success.

In this special issue of the International Journal of Environmental Research and Public Health, we have brought together research that debates and provides innovative health workforce solutions directed towards meeting population needs, mainly through integrated solutions or models of care. We have also included papers that cover challenges at an education or regulatory level. This special issue, entitled “The Future Health Workforce: Integrated Solutions and Models of Care”, features a compelling range of research that spreads across the health professions, including medicine, nursing, dentistry, and allied health. This edition embraces quantitative as well as qualitative research approaches, as well as methodological pluralism and a rapid review. A hallmark of each article is methodological rigor, and we are particularly pleased to have included research conducted with health workforce groups dealing with different conditions in a range of contexts and countries including the USA, the UK, Canada, Australia, Sweden, South Korea, Japan, China, and Brazil. This special issue features 13 papers.

The first research paper, from a multidisciplinary team of researchers based in the Rural Clinical School in the Faculty of Medicine at the University of Queensland in Australia, provides a theory that assists us to understand factors that affect doctors in choosing a generalist or specialist medical career [1]. Belinda O’Sullivan and colleagues’ theory shows us that the decision-making process involves multi-level contextual factors that intersect with triggers that produce a career preference. Both clinical and context-specific exposures, as well as attributes, skills, norms, and the status of generalist and specialist fields affects choice. These factors combine with doctors’ interests and expectations, including their professional values, and perceptions about socio-economic and lifestyle rewards. It is interesting to note too, that these factors and considerations intersected with social circumstances, most especially gender and life stage.

The second article reports research conducted with the health services management workforce in China [2]. The starting point for the study by Zhanming Liang and colleagues is the fact that the traditional recruitment approach relied on clinical performance and seniority, which provided little incentive to improve competencies. The study utilised validated management competency assessment tool that was administered to directors and deputy directors of medical services (*n* = 295) in three categories of hospitals. The survey revealed that the informal and formal education received by medical leaders in these Chinese hospitals has not been effective in developing the required medical and leadership management competencies. This provides a basis for recommendations regarding health system and higher education strategies to improve the management competencies of clinical leaders in China. 

We then turn to a thematic analysis of Twitter data and newspapers extracted through a search for new forms of team work in the health and social care of older people in response to the COVID-19 pandemic [3]. The study conducted out of University College, Dublin in Ireland, identified rapid transformations in ways of working, including innovations in telehealth, and in using online platforms to facilitate team meetings. Interestingly, much of the change was attributed to goodwill as a response to the pandemic.

Catherine Cosgrave’s study addresses chronic workforce shortages and unmet health care needs in rural and remote communities in Australia [4]. The findings from this qualitative study (semi-structured interviews with 74 executive staff, managers, and allied health professionals) revealed factors influencing the recruitment and retention of allied health professionals in rural public sector health services in Australia. The study emphasises the value of a ’whole-of-community approach’ that supports individual allied health professionals and their families to adjust to a new place and develop a sense of belonging in a new community. 

The next paper in this special issue reports a national cross-sectional study of faculties supporting general medical practitioners (GPs) [5]. Matthew McGrail and Belinda O’Sullivan report data obtained from an annual national cross-sectional survey of doctors in Australia conducted between 2008 and 2017. The survey revealed that GPs with fellowship of a rural faculty, were more likely to use advanced skills, especially procedural skills, compared with standard GPs. Membership in a rural faculty was also associated with significantly improved geographic distribution. Thus, the rural faculties were found to be critical in building and sustaining a general medical practice workforce that is better able to respond to health needs in smaller, often isolated, communities. 

The following paper takes us to research conducted on an innovative model of workers’ healthcare assistants by a group of Portuguese researchers in Brazil [6]. This study presented and validated the Workers’ Healthcare Assistance Model (WHAM) which includes an interdisciplinary approach to health risk management. The study was conducted between 2011 and 2018 in a workers’ occupational health center in the oil industry in Brazil. The study of a sample of workers (*n* = 965) showed a sustainable return on investment, covering workers with heart disease and diabetes. The study concludes that this model of workers’ healthcare assistants is capable of enhancing workers’ health in companies, while reducing costs for employers and improving workers’ quality of life within the organisation.

Luis Miguel Dos Santos has investigated reasons behind the shortage of public health, social work, and psychological counselling professionals who can provide multilingual services to minority groups and foreign residents in South Korea [7]. This fascinating study explored why graduates and professionals with multilingual skills in these three professions decided to leave their professional fields for the hospitality and business service sectors, particularly for those who completed their initial training at a university outside Korea. Twelve professionals were interviewed in depth, based on an approach consistent with social cognitive career theory. The results indicated that public health, social work, and psychological counselling services-related positions were not available, and that there was a lack of career development skills amongst these graduates who were working in fields such as tourism (such as a social worker working as a car valet) and marketing. 

The next paper in this special issue investigated the future of careers for public-health professionals with training in climate change based on analysis of 16 years’ worth of job postings and a survey with prospective employers [8]. Heather Krasna and colleagues from the Mailman School of Public Health at Columbia University in the USA conducted this study in a context where skills and competencies relevant to climate change have been incorporated into the curricula of schools of public health in Europe and Australia. They discovered that current employers value knowledge of fields such as climate mitigation and adaptation, climate-health justice, effects of climate on health, health impact assessment, risk assessment, pollution-health consequences and causes, geographic information system (GIS) mapping, communication, finance and economics, policy analysis, systems thinking, and interdisciplinary understanding. The study found that the current job market for public-health professionals with training in climate change appears small and may grow in the next 5–10 years.

Innovative health workforce solutions were needed for the Swedish mental health workforce due to the recent refugee crisis. Sandra Gupta and colleagues from Uppsala University Sweden explored the experiences of mental health workers towards new training solutions to effectively manage unaccompanied refugee minors [9]. They suggest that dealing with suicidal ideation can be challenging and feelings of helplessness can occur. They suggest that collaboration between agencies and key stakeholders as essential when targeting refugee mental health in a stepped care model to assist the mental health workforce.

The next paper from Sierras-Davo and colleagues based in Spain and Greece discusses how you can transform the future healthcare workforce across Europe through improvement science [10]. They evaluate the experience of European nursing students after an intensive one-week summer programme conducted in 2019 at the University of Alicante in Spain. Based on the findings from the study, values like compassion, respect, or empathy were identified as key elements of care. Furthermore, promoting international students’ networking emerged as the key to creating a positive provision for change and the generation of improvement initiatives. They suggest that building a healthcare improvement science culture may provide future healthcare professionals with critical thinking skills and the resources needed to improve their future work settings.

Yuki Ohara and colleagues based in Japan discuss an interesting paper on job attractiveness and job satisfaction of dental hygienists based on the 2019 Japanese dental hygienists survey [11]. Using a nationally representative data set of 7869 working dental hygienists, they analyse seven items of job attractiveness, 14 items of job satisfaction, and 13 items of request to improve the working environment. They implement item response theory and structural equation modelling (SEM) in the analysis. They identify that dental hygienists preferred national qualifications more than income stability. The SEM also showed that job satisfaction consisted of two factors, ‘value for work’ and ‘working environment’, as did job attractiveness, with ‘contribution’ and ‘assured income’. Finally, they suggest that improving job satisfaction and work environments could help to improve the employment rate of dental hygienists, which could positively influence patient care.

A very interesting commentary is featured as the penultimate article, titled Broken Promises to the People of Newark. Franklin et al [12] discuss the relationship between organised medicine, state and local leaders, and the people of Newark. The authors emphasise that among medical schools, Rutgers New Jersey Medical School’s commitment to Newark is meaningfully unique. This social contract between the medical school and the people of Newark is identified through the portrayal of historical events which led to the establishment and development of the medical school.

We round out this special issue with a rapid review of contemporary techniques and practices in oral health workforce modelling, conducted by a team of researchers from England and Australia [13]. Workforce modelling is used to inform health workforce planning through examination of the current and future supply of professionals against the need and demands of a population. The rapid review included 23 studies from 15 different countries. The study identifies that dentists were the sole oral-health workforce group modelled in 13 studies; only five studies included skill-mix (allied dental personnel) considerations. Furthermore, the most common application of modelling was a workforce to population ratio or a needs-based demand weighted variant. Nearly all studies presented weaknesses in modelling process due to the limitations in data sources and/or nonavailability of necessary data to inform oral health workforce planning. Skill-mix considerations in planning models were also limited to horizontal integration within the oral health professions. This timely study identifies that planning for the future oral health workforce is heavily reliant on quality data being available for supply, demand, and needs models. Integrated methodologies that expand skill-mix considerations and account for uncertainty are essential for future planning exercises.
